# Study on the effect of the treatment of *Periplaneta americana* L. extract Ento-B by Dinitrochlorobenzene combined with acetic acid induced UC in rats

**DOI:** 10.1590/ACB360102

**Published:** 2021-02-15

**Authors:** Sheng-Shuai Liu, Yu-Sheng Xu, Ahunova Hilola, Nong Zhou, Miao He, Cheng-Gui Zhang, Heng Liu

**Affiliations:** 1 MM. Dali University – Yunnan Provincial Key Laboratory of Entomological Biopharmaceutical R&D - Yunnan, China.; 2 MD. Hunan Agricultural University – Changsha, Hunan, China.; 3 MD. Namangan State University – Life Sciences Faculty – Uzbekistan, China.; 4 MD. Chongqing Three Gorges University – College of Biology and Food Engineering – Chongqing Engineering Laboratory – Chongqing, China.; 5 MD. Dali University – National-Local Joint Engineering Research Center of Entomoceutics – Yunnan, China.; 6 MD. Dali University – Yunnan Provincial Key Laboratory of Entomological Biopharmaceutical R&D – Yunnan, China.

**Keywords:** Periplaneta, Dinitrochlorobenzene, Ulcerative Colitis, Sulfasalazine, Rats

## Abstract

**Purpose:**

To study the Periplaneta americana L. extract Ento-B on the treatment of chronic ulcerative colitis induced by 2,4-dinitrochlorobenzene and acetic acid in rats and to explore its primary mechanism of action.

**Methods:**

Using 2,4-dinitrochlorobenzene combined with acetic acid to induce chronic ulcerative colitis (chronic UC) in rats. The sulfasalazine (400 mg/kg) and Ento-B (200 mg/kg, 100 mg/kg,50 mg/kg) were given by intragastric administration and the effect was evaluated according to the disease activity index (DAI) score, colon mucosal injury index (CMDI) score, histopathological score (HS) and the serum levels of Interleukin-4(IL-4), Interleukin-10(IL-10), Tumor necrosis factor-α(TNF-α), Malondialdehyde(MDA), Superoxide dismutase(SOD) and Inducible nitric oxide synthase(iNOS.)

**Results:**

Compared with the model group, all doses of Ento-B could reduce the score of CMDI (p < 0.05), HS(p < 0.05 or p < 0.01), significantly increased the expression of IL-4, IL-10, SOD (p < 0.01) and decreased the levels of TNF-α, MDA, iNOS in serum of UC rats, significantly improving the degree of colon lesionsin UC rats.

**Conclusions:**

Ento-B may play an important role in the treatment of ulcerative colitis induced byUC rats. The mechanism may be related to the increased expression of IL-4, IL-10, SOD and reduced expression of TNF-α, MDA, iNOS.

## Introduction

Ulcerative colitis (UC) is a common chronic nonspecific intestinal disease with unknown pathogenesis. Its main clinical manifestations are diarrhea, abdominal pain, mucous purulent and bloody stool, acute and severe complications^1^. Currently, aminosalicylic acid, glucocorticoid and immunobiological treatment are mainly used in clinics, but the use is limited because of unstable curative effect, high toxicity and strong dependencey^2^. *Periplaneta americana* L., commonly known as “cockroach”, is an insect of the genus *Blattella* of the family Cockroach, which is often used in medicine as dry or fresh insects, and was first recorded in the classic of *Shennong’s Classic of Material Medical* 3. Modern medical research has found that *Periplaneta americana* L. has the functions of anti-tumor, enhancing immunity, protecting liver, antibacterial, anti-inflammation and analgesia, tissue repair and so on^4^^–^ 9. The previous study of the group found that the active ingredient Ento-B extracted from *Periplaneta americana* L. by new technology has the effect of promoting coagulation and hemostasis^10^. The main purpose of this study was to investigate the effect of Ento-B on ulcerative colitis induced by 2,4-dinitrochlorobenzene (DNCB) combined with acetic acid in rats and to explore its mechanism, so as to provide effective data support for the development of *Periplaneta americana* L. extract Ento-B as a drug for the prevention and treatment of ulcerative colitis.

## Methods

Seventy SD male rats (SPF grade), 180-220 g, were purchased from Sichuan Chengdu Dashuo Experimental Animal Co., Ltd., License No.: SCXK (Sichuan) 2015-030, batch number: 20150624; IVC system (18 C, 50% RH) adaptive feeding in the Experimental Animal Center of Dali University.

### Drugs

Sulfasalazine (SASP), Shenzhen Regent Biochemical Technology Co., Ltd., batch number: 20140925; Ento-B extract of *Periplaneta americana* L. is provided by the Institute of insect Biomedicine, Dali University, batch number: 20140438.

### Instruments

Electronic balance, METTLER TOLEDO, model: ML204/02; ultraviolet-visible spectrophotometer, PERSEE ANALYTICS, model: T6 New Century; enzyme meter, Austria Anthos Company, model: 201; electric blast drying oven, Beijing ever bright medical treatment instrument Co., Ltd., model: 101E.

### Reagents

2,4-dinitrochlorobenzene (AR, Shandong West Asia Chemical Industry Co., Ltd., batch number: P8585); acetone (AR, Sichuan Xilong Chemical Co., Ltd, Batch number: 20140528); acetic acid (AR, Ruijin Ruijinte Chemical Co., Ltd., Batch number: 20130604); rat interleukin 4 (IL-4), rat interleukin 10 (IL-10) ELISA kit (Neobioscience Co., Ltd., batch number: R150929-002a); tumor necrosis factor-ɑ (TNF-ɑ) ELISA kit (Neobioscience Co., Ltd., batch number: 20151125); occult blood kit (Nanjing Jiancheng Bioengineering Institute, batch number: 20150424); malondialdehyde (MDA) kit (Nanjing Jiancheng Bioengineering Institute, batch number: 20151125); superoxide dismutase (SOD) kit (Nanjing Jiancheng Bioengineering Institute, batch number: 20151226); inducible nitric oxide synthase (iNOS) kit (Nanjing Jiancheng Bioengineering Institute, batch number: 20151221).

### Experimental methods

#### Establishment of the model

The model is established with reference to the research of Wang *et al*.^11^. Seventy healthy SD rats of SPF grade were used, 10 rats were randomly selected as a normal control group and the other 60 rats were numbered and their backs were shaved. Evenly smear the film on the shaved area of the back of rats with 2.0% DNCB acetone solution, 0.3 mL/time, once a day for 14 consecutive days (during this period, animals were fed normally). Pressing 0.25 mL/only on the 15th day, 0.1% DNCB ethanol (50% concentration) was intragastrically administered to rats (fasting without water for 24 hours before intragastric administration). On the 16th day, 6% acetic acid solution (0.7 mL) was administrated in the same way by intragastric injection on the previous day. The accurate time was set for 10 seconds, then flushed with 3 mL of normal saline. To establish a chronic rat UC model induced by DNCB combined with acetic acid. The normal group was operated in the above way and the modeling reagent was replaced by normal saline.

#### Grouping and mode of administration

On the 7th day after the establishment of the model, the disease activity index was carried out according to Hamamoto *et al*.^12^ (Table 1). The remaining model rats were divided into 5 groups by stratified random sampling after excluding the rats with very mild inflammation: model group, sulfasalazine group (400 mg/kg), high, middle and low dose Ento-B groups, with 10 rats in each group. Only the normal and the model groups were given normal saline (2.5 ml/kg), the other groups were given the same volume of drugs by intragastric administration. The rats in each group were given intragastric administration on the 1st day after grouping for 14 consecutive days and the disease activity index was scored on the 1st, 7th and 14th day after administration. After the last administration, fasting could not abstain from water for 24 hours. Rats were anesthetized with 10% chloral hydrate solution. Abdominal aorta blood was taken and placed at 4 ºC for 4 h. The upper serum was collected by 3000 r/min centrifugation for 10 min. According to the instructions of the kit, the expression levels of TNF-α, IL-4, IL-10 and the activities of MDA, SOD and iNOS were detected. The liver, spleen and thymus were weighed, and calculated organ index (organin dex = organ weight/body weight × 100%). The intestinal cavity was cut along the mesenteric margin, the feces were rinsed with normal saline and the colon was weighed. The colonic mucosal injury index (CMDI) was scored according to the criteria of Ekström *et al*.^13^ and Luk *et al*.^14^. The diseased colon was fixed with 40 mg/mL formaldehyde, and the pathological sections were made and the histopathological score (HS) was evaluated according to the standard of Ekström *et al*.^13^.

**Table 1:** Evaluation of disease activity index (DIA).

**DIA score**	**Stool consistency**	**Occultblood test**	**Weight loss (%)**
0	Normal	Negative(–)	< 1
1	Normal sparse stool	Weak positive(+)	1–5
2	Sparse stool	Positive(++)	5–10
3	Sparse stool diarrhea	Strong positive (+++)	10–15
4	Diarrhea	Bloody stool	≥ 15

#### Data processing

The statistical software SPSS 17.0 is used to carry out the test. Each group of experimental data is represented by *x* ± *s*, the data, in accordance with the normal distribution, are tested by variance test and the data that do not conform to the normal distribution are tested by rank-sum test, there was a statistical difference in terms of p < 0.05 or p < 0.01.

### Experimental results

#### Observations on the general condition of rats

The rats in the normal group showed the hair was smooth and white, the reaction was flexible, the activity was normal, the bodyweight had no obvious change, the diet and drinking water were normal and the feces were hard and shaped. The rats in the model group showed varying degrees of mental malaise, loose, yellowish, dull hair, slow reaction, rapid weight loss, a significant reduction in food intake, arched back, gathering and lazy movement. It is also accompanied by symptoms such as bloody stool or diarrhea, feces adhesion to the anus and so on. One week after the end of the model, the mental state of the rats recovered to a certain extent, sparse stool, blood in the stool and weight gain; after treatment with SASP, the mental state, independent movement ability and bodyweight of UC rats recovered quickly and the number of stools decreased, the sparse stool disappeared and the feces formed. After intragastric installation of different doses of Ento-B, all the indexes of UC rats were recovered to a certain extent, in which the body weight of rats in the high dose Ento-B group recovered quickly, the phenomenon of sparse stool disappeared, the feces basically formed, the independent activity increased and the mental state was good.

#### Effect of Ento-B on DAI score of UC rats

**Figure 1:**
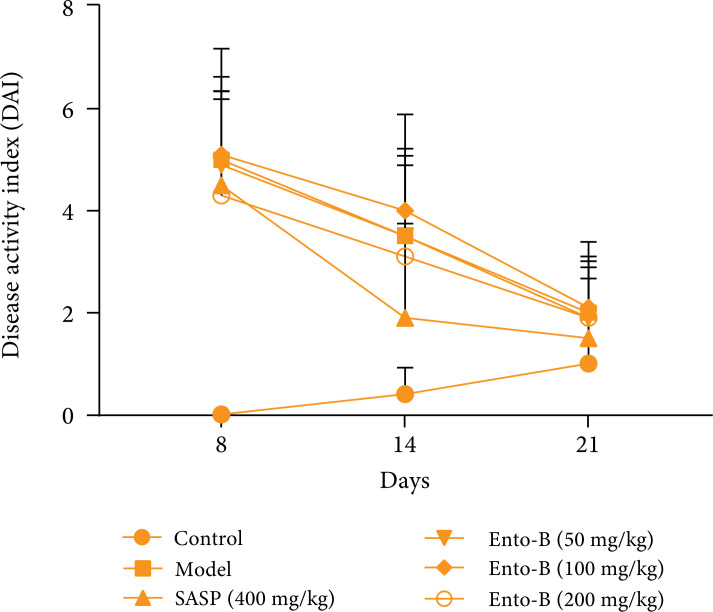
Effect of Ento-B on DAI in UC rats.

After the establishment of the model, the rats showed varying degrees of anorexia, lazy movement, weight loss, diarrhea, bloody stool and so on. After grouping, compared with the normal group, the DAI scores of rats in the model group, SASP group and Ento-B group were significantly higher than those in the normal group and the difference was statistically significant (p < 0.01). With the extension of time, the DAI of the model group decreased gradually, reflecting the self-healing process of UC model. On the 21st day after the establishment of the model, the DAI score of the model group was higher than that of the normal group (p < 0.05). The results are shown in Fig. 1.

#### Effect of Ento-B on immune organ index of UC rats

**Figure 2:**
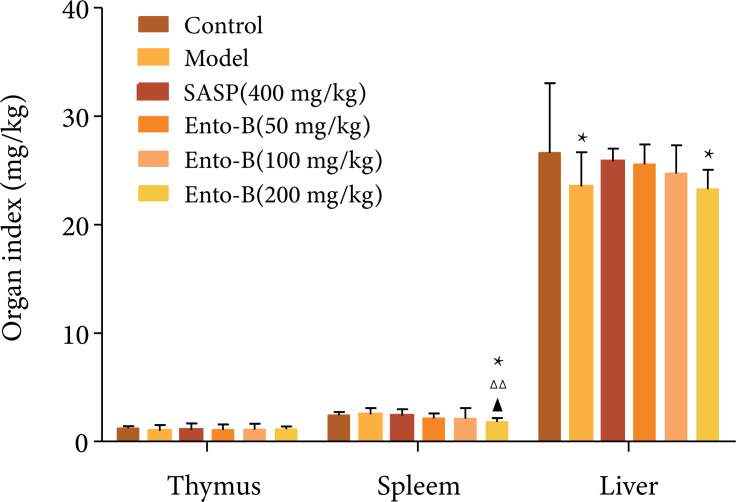
Effects of Ento-B on organ index of UC rats.

Compared with the normal group, the liver index in the model group decreased significantly (p < 0.05). Compared with the model group, there was no significant difference in thymus index, spleen index and liver index between SASP group and Ento-B middle and low dose groups (p > 0.05). The results are shown in Fig. 2.

#### Effects of Ento-B on colonic index, length, aspect ratio and CMDI score in UC rats

**Figure 3:**
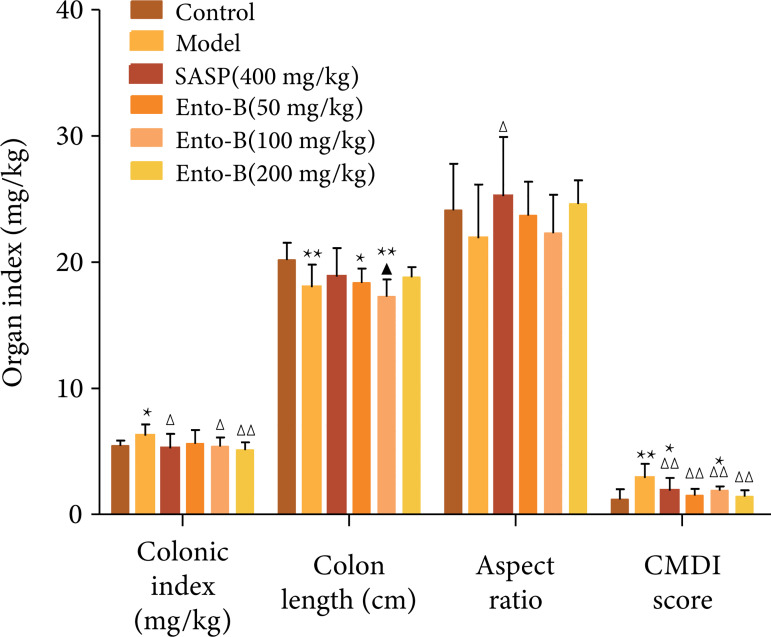
Effects of Ento-B on colon of UC rats.

Compared with the normal group, the colon index of UC rats in the model group was significantly increased, the colon length was significantly decreased and the CMDI score was significantly increased (p < 0.01). Compared with the model group, the colon CMDI score of UC rats decreased significantly after treatment in each group (p < 0.01). The results are shown in Fig. 3.

#### Effects of Ento-B on the expression of MPO in colonic tissue of UC rats

**Figure 4:**
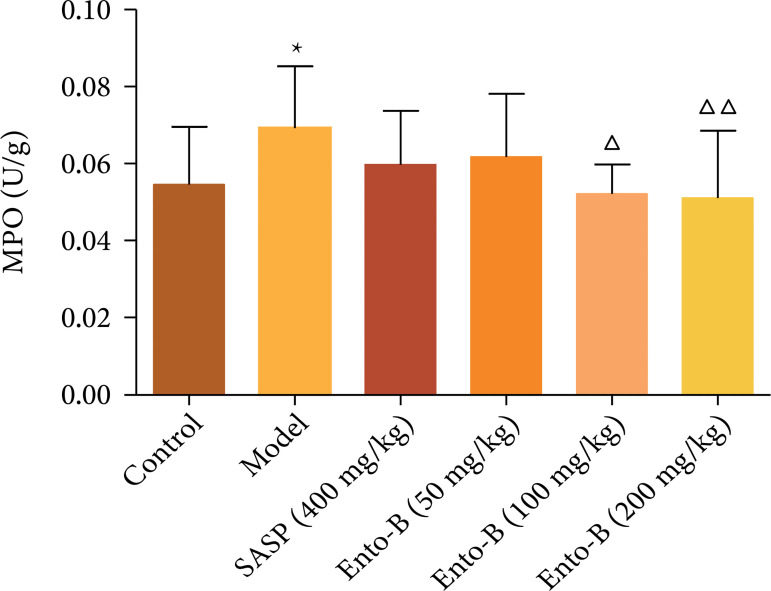
Effects of Ento-B on the expression of MPO in colonic tissue of UC rats.

Compared with the normal group, the expression of MPO in the colon tissue of the model group increased (p < 0.05). Compared with the model group, the expression of MPO in colonic tissue of UC rats decreased in varying degrees after treatment and the high and middle doses of Ento-B decreased significantly (p < 0.05 or p < 0.01). The results are shown in Fig. 4.

#### Effects of Ento- B on the expression of IL- 4, IL- 10, TNF- α, iNOS, MDA and SOD in serum of UC rats

**Figure 5:**
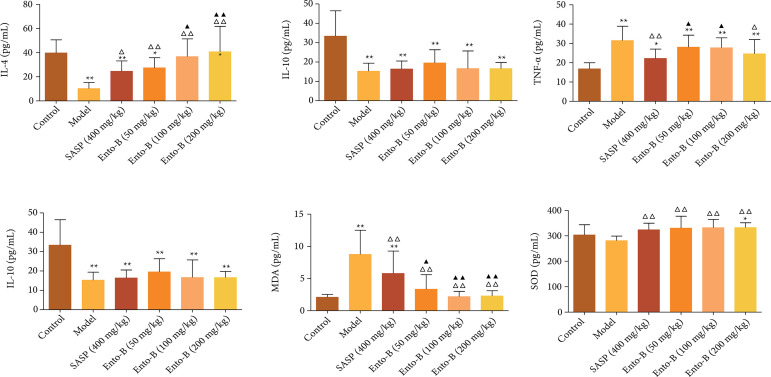
Effects of Ento-B on the expression of IL-4, IL-10, TNF-α, iNOS, MDA and SOD in serum of UC rats.

Compared with the normal group, the expression of IL-4, IL-10 and SOD in the serum of the model group decreased significantly, while the expression of TNF-*α*, MDA and iNOS increased significantly (p < 0.01). Compared with the model group, after treatment, the expression of IL-4 and SOD in the serum of UC rats was significantly increased (p < 0.05 or p < 0.01), while the expression level of MDA in the serum of UC rats was significantly decreased (p < 0.01). After oral administration of SASP and high dose Ento-B, the expression of TNF-*α* in the serum of UC rats was significantly decreased (p < 0.01 or p < 0.05) and it was dose dependent. The results are shown in Fig 5.

#### Effect of Ento- B on HS score of colonic tissue in UC rats

**Figure 6:**
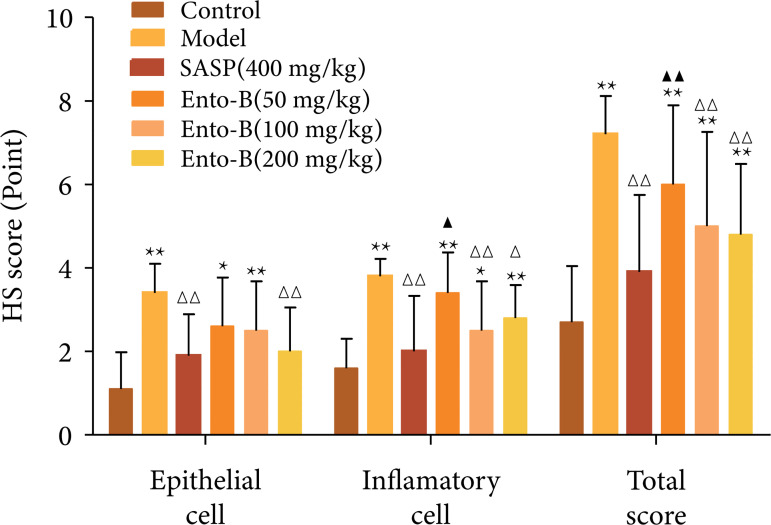
Effects of Ento-B on the expression of IL-4, IL-10, TNF-α, iNOS, MDA and SOD in serum of UC rats.

**Figure 7:**
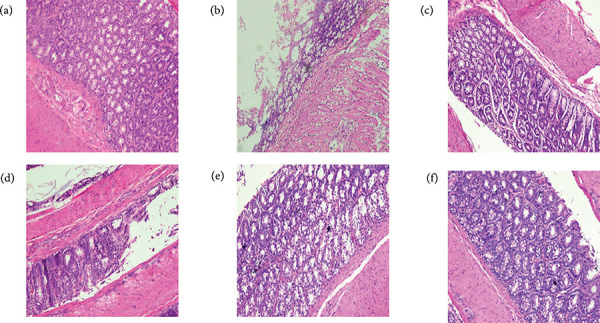
Effects of Ento-B on colonic tissue of UC rats (HE 10 × 20). (**a**) Colonic tissue sections of rats in the normal group. (**b**) Colonic tissue sections of rats in the model group. (**c**) Colonic tissue sections of rats in SASP group. (**d**) Colonic tissue sections of rats in the low dose group of Ento-B. (**e**) Colonic tissue sections of rats in the middle dose group of Ento-B. (**f**) Colonic tissue sections of rats in the high dose group of Ento-B.

In the normal group, the colonic mucous layer, submucosa, muscular layer and serous layer were clear and intact, the epithelial cells were intact, the glands were arranged neatly, the recess was normal and there was no congestion and edema and no inflammatory cell infiltration. The scores of epithelial cells, inflammatory cells and HS in the colonic tissue of the model group were significantly higher than those of the normal group (p < 0.01). Compared with the model group, SASP and high-dose Ento-B could significantly reduce the scores of colonic epithelial cells, inflammatory cell infiltration and total HS scores in UC rats (p < 0.05 or p < 0.01). The infiltration score of inflammatory cells and the total score of HS in colonic tissue of UC rats were significantly decreased by intragastric administration of middle and low doses of Ento-B (p < 0.01) and the total scores of colonic epithelial cells and HS in colonic tissue were dose-dependent with Ento-B. The results are shown in Figs. 6 and 7.

## Discussion

2,4-dinitrochlorobenzene is a kind of semi-antigen chemical, which induces delayed response in intestinal mucosa of rats after repeated sensitization, resulting in imbalance of Th1/Th2 immune cells^15^. Acetic acid can increase intestinal mucosal vascular permeability, activate kinin, interfere with coagulation, initiate inflammation, form local inflammatory lesions and achieve the combination of immune abnormalities and local inflammatory lesions, which is similar to that of human ulcerative colitis^16^. In this experiment, DNCB combined with acetic acid was used to induce chronic ulcerative colitis in rats. Acute pathological manifestations could be seen after acetic acid treatment and the inflammation changed from acute to chronic and persisted one week later. During the continuous period of chronic inflammation, the body weight of the rats gradually increased and the DAI score decreased gradually, but the feces were still thin and bloody. After the rats were killed, the colonic lumen became thicker, the colonic mucosa showed punctate ulcer or thickening and the colonic epithelial cells in the model control group were damaged and a large number of inflammatory cells infiltrated in the mucous membrane and submucosa, which was an ideal model of chronic UC. After the establishment of the model, the rats showed symptoms such as diarrhea, bloody stool and weight loss, and the DAI score increased, but the DAI score decreased after one week and there was no significant difference among the model groups. After the treatment of Ento-B, compared with the model group, the physical signs and inflammatory indexes of UC rats were improved in varying degrees, suggesting that Ento-B, the extract of *Periplaneta americana* L., has an obvious relieving effect on UC rats by intragastric administration. Tumor necrosis factor-ɑ is recognized as a pro-inflammatory factor and immunomodulatory factor that mediates the occurrence and development of UC. It can induce a variety of cell proliferation and apoptosis, activate vascular endothelial cells, express a variety of cytokines and adhesion molecules and trigger a series of important inflammatory responses^17^. The serum TNF-α of rats in the model group increased significantly. After treatment with Ento- B, the expression of TNF-α was inhibited and the inflammation was relieved. The results in the high dose group were similar to those in the SASP group and almost the same as those in the normal group. Interleukin 4and interleukin 10are mainly secreted by Th2 cells and have the characteristic of inhibiting inflammation. It was found that the expression of IL-4 and IL-10 in UC patients decreased in remission stage, while the content of IL-4 and IL-10 in model control group decreased significantly, which was consistent with that of UC patients in remission stage^2^. The levels of IL-4 and IL-10 in each group of Ento-B were significantly increased, which showed the effect of enhancement and inhibition of inflammation. In the inflammatory reaction, the expression of MDA and SOD is often used as one of the indicators to judge oxidative damage. Lipid peroxidation of superoxide ions during inflammation can increase the level of MDA, accelerate the cross-linking of nucleic acid and protein, change cell function, induce cell injury and apoptosis and aggravate inflammation, while the increased expression of SOD can remove superoxide ions and slow down inflammation^18^. Ento- B could significantly increase the expression of SOD, inhibit the production of MDA and reduce oxidative damage. Nitric oxide is an important regulator of information transmission between cells. When inflammation occurs, it can induce the synthesis of iNOS, promote inflammatory response and promote vasodilation, edema and cytotoxicity. The high dose group of Ento-B could significantly inhibit the expression of iNOS, relieve edema and decrease the scores of CMDI and HS in model rats.

## Conclusions

Ento-B, the extract of *Periplaneta americana* L., has a therapeutic effect on chronic UC induced by DNCB combined with acetic acid. It can regulate the expression of immune factors, inhibit inflammation and improve the degree of the colonic lesion. The mechanism may be related to the increase of IL-4, IL-10, SOD, and the decrease of TNF- α, MDA and iNOS expression in rats.
